# Supplementary material for: CT dose reduction factors in the thousands using X-ray phase contrast
Source: Sci Rep. 2017 Nov 21;7:15953. doi: 10.1038/s41598-017-16264-x (PMC5698457; doi:10.1038/s41598-017-16264-x)
Supplement: Supplementary file 1 — Supplementary Information [file 41598_2017_16264_MOESM1_ESM.pdf]

Supplementary Materials for

CT dose reduction factors in the thousands using X-ray phase contrast

Authors: Marcus J. Kitchen[1]*, Genevieve A. Buckley[1], Timur E. Gureyev[2,3,1], Megan J. Wallace[4,5], Nico Andres-Thio[6,7], Kentaro Uesugi[8], Naoto Yagi[8] and Stuart B. Hooper[4,5].

Affiliations:

[1]School of Physics and Astronomy, Monash University, Melbourne, 3800, Australia.

[2]ARC Centre of Excellence in Advanced Molecular Imaging, School of Physics, University of Melbourne, Parkville, 3052, Australia.

[3]School of Science and Technology, University of New England, Armidale, 2351, Australia.

[4]The Ritchie Centre, Hudson Institute for Medical Research, Melbourne, 3168, Australia.

[5]Department of Obstetrics and Gynaecology, Monash University, Melbourne, 3800, Australia.

[6]School of Engineering, University of Melbourne, Parkville, 3052, Australia.

[7]School of Mathematics and Statistics, University of Melbourne, Parkville, 3052, Australia.

[8]Japan Synchrotron Radiation Research Institute (JASRI/SPring-8), 1-1-1 Kouto, Sayo, Hyogo 679-5198, Japan.

*Correspondence to: Marcus.Kitchen@monash.edu.

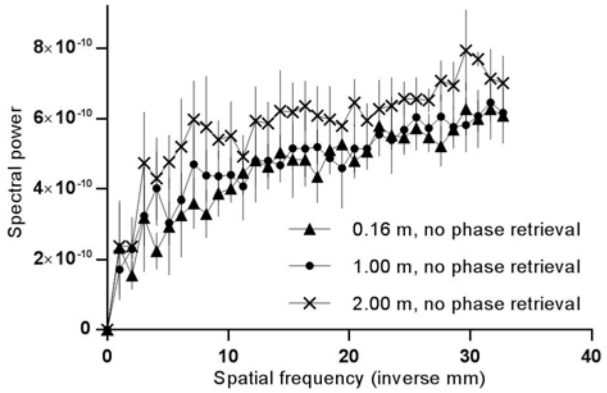

Figure S1. Power spectrum from CT data showing image noise is essentially constant as a function of free space propagation distance. Image noise is characterised by the spectral power at different spatial frequencies; here it is shown that additional free space propagation between the sample and detector does not significantly increase image noise in the reconstructed CT slices (64×64 pixel ROIs). Uncertainties given by the standard deviation of repeated measurements on five CT slices.

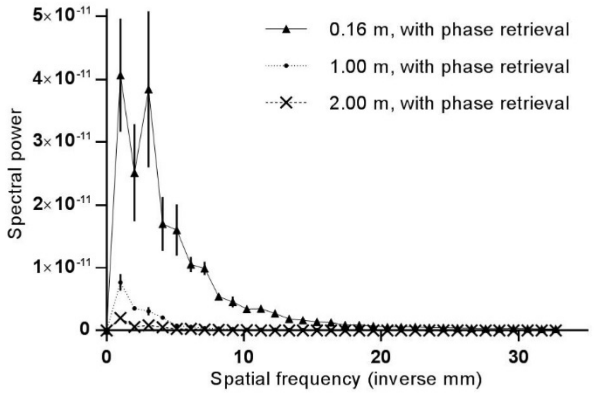

Figure S2. Power spectrum from CT showing noise suppression due to phase retrieval. Here we see the effect of phase retrieval, causing a decrease in spectral power for the highest spatial frequencies and thus suppressing image noise (64×64 pixel ROIs). Uncertainties are given by the standard deviation of repeated measurements on 5 CT slices.

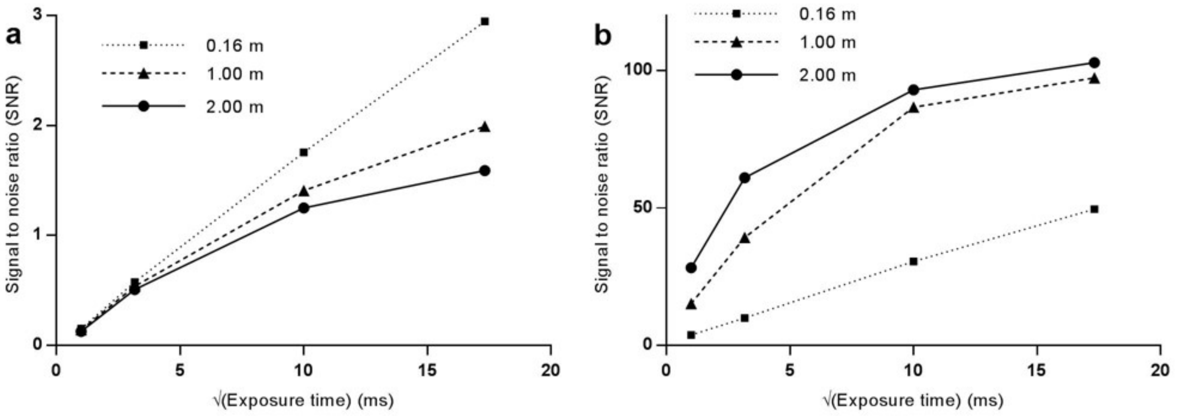

Figure S3. SNR measures plotted against the square root of exposure time a) without and b) with phase retrieval. At the shortest distance we see the expected linear proportionality. As the distance is increased, phase contrast creates artefacts that reduce the SNR. Phase retrieval (b) removes the bulk of the phase contrast and other artefacts. Uncertainty bars are too small to see on these scales.

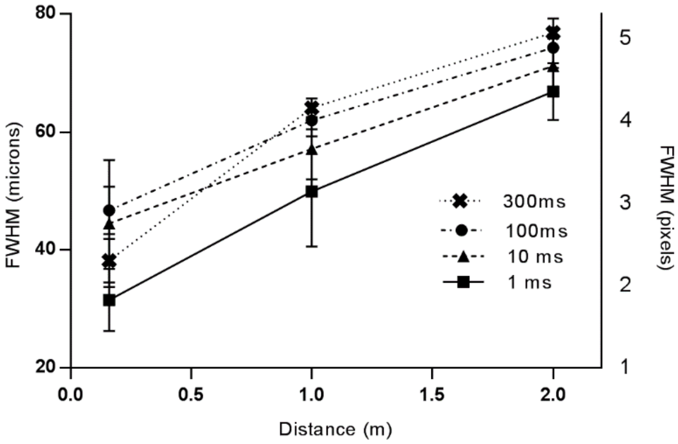

Figure S4. Spatial resolution of CT reconstructions. Uncertainties are represented by the standard deviation over n = 10 measurements. Exposure times were 1 ms (squares, solid line), 10 ms (triangles, dashed line), 100 ms (circles, dash-dotted line), and 300 ms (crosses, dotted line). This apparent loss in resolution results partly from imperfect coherence and likely the deliberate assumption of the object being comprised of water only in the phase retrieval, as described in the Materials and Methods section. Neither effect should affect the SNR calculations.
